# (5,5′-Dicarboxy­biphenyl-2,2′-dicarboxyl­ato-κ^2^ *O* ^2^,*O* ^2′^)bis­(1,10-phenanthroline-κ^2^ *N*,*N*′)zinc(II) dihydrate

**DOI:** 10.1107/S1600536808015742

**Published:** 2008-06-07

**Authors:** Rui-Zhan Chen, Fei-Jun Guo, Fan-Lei Meng

**Affiliations:** a College of Chemistry, Changchun Normal University, Changchun 130032, People’s Republic of China; b Changchun Institute of Applied Chemistry, Chinese Academy of Sciences, Changchun Center of Mass Spectrometry, Changchun 130022, People’s Republic of China

## Abstract

In the title compound, [Zn(C_16_H_8_O_8_)(C_12_H_8_N_2_)_2_]·2H_2_O, the Zn^II^ atom is located on a twofold rotation axis and is six-coordinated by two O atoms from a 5,5′-dicarboxy­biphenyl-2,2′-dicarboxyl­ate ligand and four N atoms from two 1,10-phenanthroline mol­ecules in a distorted octa­hedral geometry. The crystal structure involves O—H⋯O hydrogen bonds.

## Related literature

For related literature, see: Che *et al.* (2006 ▶); Chen *et al.* (2008 ▶); Lehn (1990 ▶); Zang *et al.* (2006 ▶).
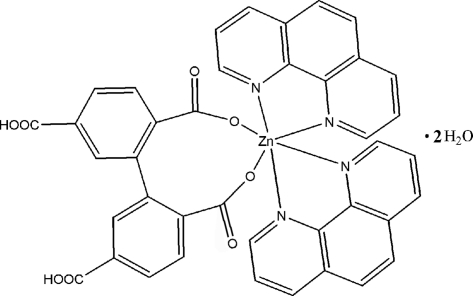


## Experimental

### 

#### Crystal data

[Zn(C_16_H_8_O_8_)(C_12_H_8_N_2_)_2_]·2H_2_O
*M* *_r_* = 790.03
Monoclinic, 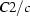

*a* = 16.901 (5) Å
*b* = 9.473 (3) Å
*c* = 22.126 (7) Å
β = 96.429 (5)°
*V* = 3520.4 (19) Å^3^
*Z* = 4
Mo *K*α radiation
μ = 0.77 mm^−1^
*T* = 293 (2) K
0.26 × 0.22 × 0.20 mm

#### Data collection

Bruker SMART APEX CCD area-detector diffractometer
Absorption correction: multi-scan (*SADABS*; Bruker, 2001 ▶) *T*_min_ = 0.817, *T*_max_ = 0.853
9664 measured reflections
3487 independent reflections
2437 reflections with *I* > 2σ(*I*)
*R*_int_ = 0.049

#### Refinement

*R*[*F* ^2^ > 2σ(*F* ^2^)] = 0.057
*wR*(*F* ^2^) = 0.124
*S* = 1.04
3487 reflections
255 parameters
2 restraints
H atoms treated by a mixture of independent and constrained refinement
Δρ_max_ = 0.31 e Å^−3^
Δρ_min_ = −0.22 e Å^−3^

### 

Data collection: *SMART* (Bruker, 2007 ▶); cell refinement: *SAINT* (Bruker, 2007 ▶); data reduction: *SAINT*; program(s) used to solve structure: *SHELXS97* (Sheldrick, 2008 ▶); program(s) used to refine structure: *SHELXL97* (Sheldrick, 2008 ▶); molecular graphics: *SHELXTL* (Sheldrick, 2008 ▶); software used to prepare material for publication: *SHELXTL*.

## Supplementary Material

Crystal structure: contains datablocks global, I. DOI: 10.1107/S1600536808015742/hy2133sup1.cif

Structure factors: contains datablocks I. DOI: 10.1107/S1600536808015742/hy2133Isup2.hkl

Additional supplementary materials:  crystallographic information; 3D view; checkCIF report

## Figures and Tables

**Table:** 

Zn1—O1	2.102 (2)
Zn1—N1	2.130 (3)
Zn1—N2	2.199 (3)

**Table:** 

O1—Zn1—O1^i^	106.16 (11)
O1—Zn1—N1^i^	98.70 (10)
O1—Zn1—N1	87.72 (10)
N1^i^—Zn1—N1	169.36 (16)
O1—Zn1—N2	162.88 (11)
N1—Zn1—N2	76.44 (13)
O1—Zn1—N2^i^	82.94 (10)
N1—Zn1—N2^i^	96.08 (12)
N2—Zn1—N2^i^	92.23 (15)

Symmetry code: (i) 
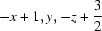
.

**Table 2:** Hydrogen-bond geometry (Å, °)

*D*—H⋯*A*	*D*—H	H⋯*A*	*D*⋯*A*	*D*—H⋯*A*
O3—H3*A*⋯O2^ii^	0.82	1.74	2.538 (3)	162
O1*W*—H1*B*⋯O4^iii^	0.86 (3)	2.24 (2)	2.966 (4)	143 (3)
O1*W*—H1*A*⋯O2	0.85 (3)	2.00 (2)	2.808 (4)	159 (4)

Symmetry codes: (ii) 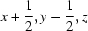; (iii) 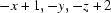.

**Fig. 1.**
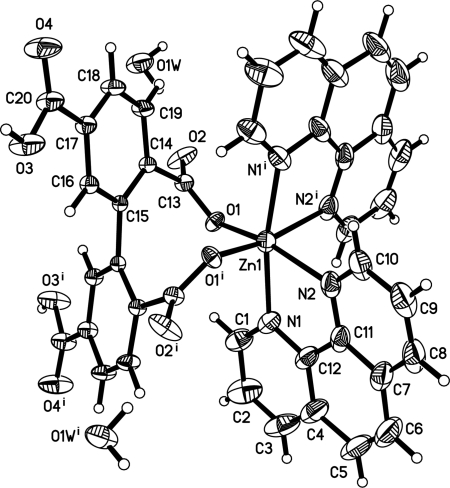
Molecular structure of the title compound. Displacement ellipsoids are drawn at the 30% probability level. [Symmetry code: (i) 1-x, y, 1.5-z.]

## supplementary crystallographic information

### Comment

In the construction of new coordination polymers, multi-carboxylates act as multifunctional organic ligands not only due to their various coordination modes, resulting from fully or partially deprotonated sites, to allow for the large diversity in topologies, but also due to the ability to act as hydrogen-bond acceptors and donors to assemble supramolecular structures (Che *et al.*,  2006; Chen *et al.*,  2008; Lehn, 1990). We chose biphenyl-2,5,2',5'-tetracarboxylic acid (H_4_bptc) as a bridging ligand, 1,10-phenanthroline (phen) as a neutral ligand, and zinc(II) as a metal center, generating the title compound. We report here its crystal structure.

In the title compound, the Zn^II^ atom, lying on a twofold rotation axis, is six-coordinated by two O atoms from one H_2_bptc ligand and four N atoms from two phen molecules in a distorted octahedral geometry (Fig. 1). The twofold rotation axis passes through the midpoint of the bond connecting two benzene rings of the H_2_bptc ligand. The bond lengths are within the normal ranges (Table 1) (Zang *et al.*, 2006). The crystal structure involves O—H···O hydrogen bonds between the carboxylate O atoms and water molecules (Table 2).

### Experimental

A mixture of ZnCl_2_.2H_2_O (0.017 g, 0.1 mmol), H_4_bptc (0.066 g, 0.2 mmol), phen (0.040 g, 0.2 mmol) and H_2_O(15 ml) in a 25 ml Teflon-lined stainless steel reactor was heated from 298 to 443 K in 2 h and a constant temperature was maintained at 443 K for 72 h. After cooling to 298 K, colorless crystals of the title compound were obtained from the reaction.

### Refinement

H atoms bonded to C atoms and carboxylate O atom were positioned geometrically and refined as riding atoms, with C—H = 0.93 and O—H = 0.82 Å and with *U*_iso_(H) = 1.2*U*_eq_(C) or 1.5*U*_eq_(O). The water H-atoms were located from a difference Fourier map and refined with a distance restraint of O—H = 0.85 (1) Å and *U*_iso_(H) = 0.064 Å^2^.

### Crystal data

**Table:**

[Zn(C_16_H_8_O_8_)(C_12_H_8_N_2_)_2_]·2H_2_O	*F*_000_ = 1624
*M**_r_* = 790.03	*D*_x_ = 1.491 Mg m^−^^3^
Monoclinic, *C*2/*c*	Mo *K*α radiation λ = 0.71073 Å
Hall symbol: -C 2yc	Cell parameters from 3487 reflections
*a* = 16.901 (5) Å	θ = 2.0–26.0º
*b* = 9.473 (3) Å	µ = 0.77 mm^−^^1^
*c* = 22.126 (7) Å	*T* = 293 (2) K
β = 96.429 (5)º	Block, colorless
*V* = 3520.4 (19) Å^3^	0.26 × 0.22 × 0.20 mm
*Z* = 4	

### Data collection

**Table:**

Bruker SMART APEX CCD area-detector diffractometer	3487 independent reflections
Radiation source: fine-focus sealed tube	2437 reflections with *I* > 2σ(*I*)
Monochromator: graphite	*R*_int_ = 0.049
*T* = 293(2) K	θ_max_ = 26.2º
φ and ω scans	θ_min_ = 1.9º
Absorption correction: multi-scan(SADABS; Bruker, 2001)	*h* = −20→18
*T*_min_ = 0.817, *T*_max_ = 0.853	*k* = −11→11
9664 measured reflections	*l* = −21→27

### Refinement

**Table:**

Refinement on *F*^2^	Secondary atom site location: difference Fourier map
Least-squares matrix: full	Hydrogen site location: inferred from neighbouring sites
*R*[*F*^2^ > 2σ(*F*^2^)] = 0.057	H atoms treated by a mixture of independent and constrained refinement
*wR*(*F*^2^) = 0.125	*w* = 1/[σ^2^(*F*_o_^2^) + (0.0505*P*)^2^ + 0.8309*P*] where *P* = (*F*_o_^2^ + 2*F*_c_^2^)/3
*S* = 1.04	(Δ/σ)_max_ < 0.001
3487 reflections	Δρ_max_ = 0.31 e Å^−^^3^
255 parameters	Δρ_min_ = −0.22 e Å^−^^3^
2 restraints	Extinction correction: none
Primary atom site location: structure-invariant direct methods	

### Special details

**Table:**

Geometry. All e.s.d.'s (except the e.s.d. in the dihedral angle between two l.s. planes) are estimated using the full covariance matrix. The cell e.s.d.'s are taken into account individually in the estimation of e.s.d.'s in distances, angles and torsion angles; correlations between e.s.d.'s in cell parameters are only used when they are defined by crystal symmetry. An approximate (isotropic) treatment of cell e.s.d.'s is used for estimating e.s.d.'s involving l.s. planes.

### Fractional atomic coordinates and isotropic or equivalent isotropic displacement parameters (Å^2^)

**Table:**

	*x*	*y*	*z*	*U*_iso_*/*U*_eq_	
C1	0.3739 (2)	0.3651 (5)	0.63923 (18)	0.0637 (11)	
H1	0.3543	0.2958	0.6634	0.076*	
C2	0.3354 (3)	0.3873 (6)	0.5804 (2)	0.0880 (16)	
H2	0.2913	0.3337	0.5656	0.106*	
C3	0.3645 (3)	0.4896 (6)	0.5454 (2)	0.0926 (17)	
H3	0.3390	0.5077	0.5067	0.111*	
C4	0.4311 (3)	0.5664 (5)	0.5667 (2)	0.0762 (14)	
C5	0.4663 (4)	0.6727 (6)	0.5317 (2)	0.0988 (19)	
H5	0.4424	0.6948	0.4929	0.119*	
C6	0.5326 (4)	0.7401 (6)	0.5538 (3)	0.106 (2)	
H6	0.5542	0.8074	0.5298	0.127*	
C7	0.5713 (3)	0.7116 (4)	0.6134 (2)	0.0776 (14)	
C8	0.6414 (4)	0.7740 (5)	0.6377 (3)	0.098 (2)	
H8	0.6659	0.8408	0.6152	0.117*	
C9	0.6744 (3)	0.7386 (5)	0.6936 (3)	0.0906 (17)	
H9	0.7222	0.7793	0.7098	0.109*	
C10	0.6361 (3)	0.6400 (4)	0.7274 (2)	0.0722 (13)	
H10	0.6593	0.6164	0.7661	0.087*	
C11	0.5368 (3)	0.6116 (4)	0.6493 (2)	0.0600 (11)	
C12	0.4662 (3)	0.5378 (4)	0.62567 (18)	0.0588 (11)	
C13	0.40756 (18)	0.1983 (3)	0.81456 (14)	0.0328 (7)	
C14	0.47901 (16)	0.1060 (3)	0.82887 (12)	0.0264 (7)	
C15	0.51756 (16)	0.0460 (3)	0.78249 (12)	0.0240 (6)	
C16	0.58975 (17)	−0.0230 (3)	0.79802 (13)	0.0299 (7)	
H16	0.6169	−0.0613	0.7676	0.036*	
C17	0.62178 (18)	−0.0353 (3)	0.85843 (14)	0.0335 (7)	
C18	0.58141 (19)	0.0192 (4)	0.90393 (14)	0.0421 (9)	
H18	0.6021	0.0094	0.9445	0.051*	
C19	0.51007 (18)	0.0882 (3)	0.88893 (13)	0.0383 (8)	
H19	0.4824	0.1235	0.9197	0.046*	
C20	0.6999 (2)	−0.1096 (4)	0.87475 (16)	0.0460 (9)	
N1	0.43688 (18)	0.4390 (3)	0.66158 (13)	0.0510 (8)	
N2	0.5682 (2)	0.5791 (3)	0.70633 (15)	0.0563 (8)	
O1	0.40798 (12)	0.2849 (2)	0.77191 (9)	0.0370 (5)	
O2	0.35134 (14)	0.1861 (3)	0.84667 (11)	0.0646 (8)	
O1W	0.33466 (18)	0.0544 (3)	0.95823 (12)	0.0704 (8)	
O3	0.73266 (15)	−0.1494 (3)	0.82746 (11)	0.0707 (9)	
H3A	0.7749	−0.1893	0.8384	0.106*	
O4	0.72855 (15)	−0.1289 (3)	0.92612 (11)	0.0786 (10)	
Zn1	0.5000	0.41820 (6)	0.7500	0.0430 (2)	
H1B	0.315 (2)	0.112 (3)	0.9823 (14)	0.064*	
H1A	0.333 (2)	0.110 (3)	0.9282 (12)	0.064*	

### Atomic displacement parameters (Å^2^)

**Table:**

	*U*^11^	*U*^22^	*U*^33^	*U*^12^	*U*^13^	*U*^23^
C1	0.053 (3)	0.081 (3)	0.058 (3)	0.011 (2)	0.009 (2)	0.017 (2)
C2	0.054 (3)	0.135 (5)	0.074 (3)	0.021 (3)	0.002 (2)	0.021 (3)
C3	0.085 (4)	0.133 (5)	0.061 (3)	0.046 (4)	0.016 (3)	0.038 (3)
C4	0.092 (4)	0.076 (3)	0.065 (3)	0.033 (3)	0.030 (3)	0.029 (3)
C5	0.145 (6)	0.088 (4)	0.072 (4)	0.041 (4)	0.049 (4)	0.042 (3)
C6	0.168 (6)	0.065 (4)	0.097 (5)	0.018 (4)	0.071 (4)	0.030 (3)
C7	0.116 (4)	0.045 (3)	0.084 (4)	0.007 (3)	0.065 (3)	0.006 (2)
C8	0.151 (6)	0.051 (3)	0.109 (5)	−0.032 (3)	0.093 (4)	−0.018 (3)
C9	0.113 (4)	0.068 (3)	0.104 (4)	−0.040 (3)	0.071 (4)	−0.032 (3)
C10	0.093 (4)	0.052 (2)	0.081 (3)	−0.023 (2)	0.047 (3)	−0.020 (2)
C11	0.083 (3)	0.035 (2)	0.070 (3)	0.007 (2)	0.044 (2)	−0.0001 (19)
C12	0.076 (3)	0.051 (2)	0.056 (3)	0.027 (2)	0.033 (2)	0.0180 (19)
C13	0.0281 (18)	0.0396 (18)	0.0312 (18)	0.0106 (14)	0.0054 (14)	0.0015 (15)
C14	0.0225 (16)	0.0314 (17)	0.0255 (16)	0.0040 (12)	0.0037 (12)	−0.0001 (12)
C15	0.0231 (16)	0.0238 (15)	0.0253 (16)	0.0002 (11)	0.0036 (12)	0.0000 (11)
C16	0.0247 (17)	0.0352 (17)	0.0304 (18)	0.0077 (13)	0.0056 (13)	−0.0018 (13)
C17	0.0262 (18)	0.0431 (18)	0.0310 (18)	0.0093 (14)	0.0018 (13)	0.0017 (14)
C18	0.039 (2)	0.063 (2)	0.0236 (18)	0.0165 (17)	0.0000 (14)	−0.0008 (16)
C19	0.0358 (19)	0.054 (2)	0.0266 (17)	0.0185 (16)	0.0097 (14)	−0.0025 (15)
C20	0.033 (2)	0.070 (3)	0.035 (2)	0.0191 (17)	0.0034 (16)	0.0049 (17)
N1	0.051 (2)	0.0519 (19)	0.052 (2)	0.0120 (16)	0.0165 (15)	0.0146 (15)
N2	0.072 (2)	0.0382 (17)	0.066 (2)	−0.0056 (17)	0.0362 (18)	−0.0067 (16)
O1	0.0319 (13)	0.0374 (12)	0.0422 (13)	0.0101 (10)	0.0063 (10)	0.0116 (10)
O2	0.0449 (16)	0.098 (2)	0.0564 (17)	0.0427 (15)	0.0294 (12)	0.0411 (15)
O1W	0.079 (2)	0.088 (2)	0.0457 (19)	0.0195 (17)	0.0126 (15)	0.0143 (15)
O3	0.0530 (17)	0.121 (2)	0.0388 (15)	0.0559 (17)	0.0072 (12)	0.0098 (15)
O4	0.0610 (19)	0.133 (3)	0.0394 (16)	0.0561 (18)	−0.0040 (13)	0.0029 (16)
Zn1	0.0470 (4)	0.0373 (3)	0.0467 (4)	0.000	0.0144 (3)	0.000

### Geometric parameters (Å, °)

**Table:**

C1—N1	1.323 (5)	C13—O2	1.254 (4)
C1—C2	1.405 (6)	C13—C14	1.496 (4)
C1—H1	0.9300	C14—C19	1.383 (4)
C2—C3	1.366 (7)	C14—C15	1.396 (4)
C2—H2	0.9300	C15—C16	1.393 (4)
C3—C4	1.378 (7)	C15—C15^i^	1.493 (5)
C3—H3	0.9300	C16—C17	1.390 (4)
C4—C12	1.399 (6)	C16—H16	0.9300
C4—C5	1.439 (7)	C17—C18	1.378 (4)
C5—C6	1.334 (7)	C17—C20	1.504 (4)
C5—H5	0.9300	C18—C19	1.379 (4)
C6—C7	1.431 (7)	C18—H18	0.9300
C6—H6	0.9300	C19—H19	0.9300
C7—C8	1.378 (7)	C20—O4	1.198 (4)
C7—C11	1.404 (5)	C20—O3	1.294 (4)
C8—C9	1.341 (7)	N1—Zn1	2.130 (3)
C8—H8	0.9300	N2—Zn1	2.199 (3)
C9—C10	1.399 (6)	O1—Zn1	2.102 (2)
C9—H9	0.9300	O1W—H1B	0.86 (3)
C10—N2	1.321 (5)	O1W—H1A	0.85 (3)
C10—H10	0.9300	O3—H3A	0.8200
C11—N2	1.348 (5)	Zn1—O1	2.102 (2)
C11—C12	1.431 (6)	Zn1—N1	2.130 (3)
C12—N1	1.357 (4)	Zn1—N2	2.199 (3)
C13—O1	1.251 (3)		
			
N1—C1—C2	122.5 (4)	C16—C15—C14	118.5 (3)
N1—C1—H1	118.8	C16—C15—C15^i^	118.7 (3)
C2—C1—H1	118.8	C14—C15—C15^i^	122.7 (3)
C3—C2—C1	118.2 (5)	C17—C16—C15	120.9 (3)
C3—C2—H2	120.9	C17—C16—H16	119.6
C1—C2—H2	120.9	C15—C16—H16	119.6
C2—C3—C4	120.8 (5)	C18—C17—C16	120.0 (3)
C2—C3—H3	119.6	C18—C17—C20	119.5 (3)
C4—C3—H3	119.6	C16—C17—C20	120.5 (3)
C3—C4—C12	117.5 (4)	C17—C18—C19	119.5 (3)
C3—C4—C5	123.8 (5)	C17—C18—H18	120.2
C12—C4—C5	118.7 (5)	C19—C18—H18	120.2
C6—C5—C4	121.3 (5)	C18—C19—C14	121.1 (3)
C6—C5—H5	119.4	C18—C19—H19	119.5
C4—C5—H5	119.4	C14—C19—H19	119.5
C5—C6—C7	121.7 (5)	O4—C20—O3	124.0 (3)
C5—C6—H6	119.1	O4—C20—C17	123.3 (3)
C7—C6—H6	119.1	O3—C20—C17	112.7 (3)
C8—C7—C11	117.4 (5)	C1—N1—C12	118.5 (4)
C8—C7—C6	124.2 (5)	C1—N1—Zn1	126.4 (3)
C11—C7—C6	118.3 (5)	C12—N1—Zn1	115.1 (3)
C9—C8—C7	120.2 (5)	C10—N2—C11	117.7 (4)
C9—C8—H8	119.9	C10—N2—Zn1	128.7 (3)
C7—C8—H8	119.9	C11—N2—Zn1	113.5 (3)
C8—C9—C10	119.3 (5)	C13—O1—Zn1	129.41 (19)
C8—C9—H9	120.3	H1B—O1W—H1A	96 (4)
C10—C9—H9	120.3	C20—O3—H3A	109.5
N2—C10—C9	122.6 (5)	O1—Zn1—O1^i^	106.16 (11)
N2—C10—H10	118.7	O1—Zn1—N1^i^	98.70 (10)
C9—C10—H10	118.7	O1^i^—Zn1—N1^i^	87.72 (10)
N2—C11—C7	122.7 (5)	O1—Zn1—N1	87.72 (10)
N2—C11—C12	117.1 (3)	O1^i^—Zn1—N1	98.70 (10)
C7—C11—C12	120.2 (5)	N1^i^—Zn1—N1	169.36 (16)
N1—C12—C4	122.4 (4)	O1—Zn1—N2	162.88 (11)
N1—C12—C11	117.8 (4)	O1^i^—Zn1—N2	82.94 (10)
C4—C12—C11	119.7 (4)	N1^i^—Zn1—N2	96.08 (12)
O1—C13—O2	123.8 (3)	N1—Zn1—N2	76.44 (13)
O1—C13—C14	118.1 (3)	O1—Zn1—N2^i^	82.94 (10)
O2—C13—C14	118.1 (3)	O1^i^—Zn1—N2^i^	162.88 (11)
C19—C14—C15	119.9 (3)	N1^i^—Zn1—N2^i^	76.44 (13)
C19—C14—C13	119.0 (3)	N1—Zn1—N2^i^	96.08 (12)
C15—C14—C13	121.0 (2)	N2—Zn1—N2^i^	92.23 (15)
			
N1—C1—C2—C3	−0.2 (7)	C16—C17—C20—O4	−176.6 (4)
C1—C2—C3—C4	1.7 (8)	C18—C17—C20—O3	−177.0 (3)
C2—C3—C4—C12	−1.5 (7)	C16—C17—C20—O3	3.8 (5)
C2—C3—C4—C5	178.1 (4)	C2—C1—N1—C12	−1.4 (6)
C3—C4—C5—C6	−177.5 (5)	C2—C1—N1—Zn1	179.3 (3)
C12—C4—C5—C6	2.2 (8)	C4—C12—N1—C1	1.6 (5)
C4—C5—C6—C7	−0.8 (9)	C11—C12—N1—C1	−176.8 (3)
C5—C6—C7—C8	177.4 (5)	C4—C12—N1—Zn1	−179.0 (3)
C5—C6—C7—C11	−1.3 (8)	C11—C12—N1—Zn1	2.6 (4)
C11—C7—C8—C9	0.1 (7)	C9—C10—N2—C11	2.0 (6)
C6—C7—C8—C9	−178.6 (5)	C9—C10—N2—Zn1	178.0 (3)
C7—C8—C9—C10	−1.2 (7)	C7—C11—N2—C10	−3.2 (5)
C8—C9—C10—N2	0.2 (7)	C12—C11—N2—C10	175.9 (3)
C8—C7—C11—N2	2.2 (6)	C7—C11—N2—Zn1	−179.8 (3)
C6—C7—C11—N2	−179.1 (4)	C12—C11—N2—Zn1	−0.8 (4)
C8—C7—C11—C12	−176.9 (4)	O2—C13—O1—Zn1	135.8 (3)
C6—C7—C11—C12	1.9 (6)	C14—C13—O1—Zn1	−42.9 (4)
C3—C4—C12—N1	−0.2 (6)	C13—O1—Zn1—O1^i^	63.6 (2)
C5—C4—C12—N1	−179.8 (4)	C13—O1—Zn1—N1^i^	−26.5 (3)
C3—C4—C12—C11	178.2 (4)	C13—O1—Zn1—N1	162.0 (3)
C5—C4—C12—C11	−1.5 (6)	C13—O1—Zn1—N2	−176.0 (3)
N2—C11—C12—N1	−1.2 (5)	C13—O1—Zn1—N2^i^	−101.6 (3)
C7—C11—C12—N1	177.9 (3)	C1—N1—Zn1—O1	−9.4 (3)
N2—C11—C12—C4	−179.6 (3)	C12—N1—Zn1—O1	171.3 (2)
C7—C11—C12—C4	−0.5 (6)	C1—N1—Zn1—O1^i^	96.6 (3)
O1—C13—C14—C19	134.7 (3)	C12—N1—Zn1—O1^i^	−82.7 (2)
O2—C13—C14—C19	−44.2 (4)	C1—N1—Zn1—N1^i^	−136.8 (3)
O1—C13—C14—C15	−40.9 (4)	C12—N1—Zn1—N1^i^	43.9 (2)
O2—C13—C14—C15	140.3 (3)	C1—N1—Zn1—N2	177.1 (3)
C19—C14—C15—C16	−4.1 (4)	C12—N1—Zn1—N2	−2.2 (2)
C13—C14—C15—C16	171.4 (3)	C1—N1—Zn1—N2^i^	−92.1 (3)
C19—C14—C15—C15^i^	172.4 (2)	C12—N1—Zn1—N2^i^	88.6 (2)
C13—C14—C15—C15^i^	−12.1 (4)	C10—N2—Zn1—O1	162.7 (3)
C14—C15—C16—C17	1.7 (4)	C11—N2—Zn1—O1	−21.1 (5)
C15^i^—C15—C16—C17	−175.0 (2)	C10—N2—Zn1—O1^i^	−73.9 (3)
C15—C16—C17—C18	1.1 (5)	C11—N2—Zn1—O1^i^	102.3 (2)
C15—C16—C17—C20	−179.8 (3)	C10—N2—Zn1—N1^i^	13.1 (3)
C16—C17—C18—C19	−1.3 (5)	C11—N2—Zn1—N1^i^	−170.7 (2)
C20—C17—C18—C19	179.5 (3)	C10—N2—Zn1—N1	−174.6 (3)
C17—C18—C19—C14	−1.1 (5)	C11—N2—Zn1—N1	1.6 (2)
C15—C14—C19—C18	3.9 (5)	C10—N2—Zn1—N2^i^	89.7 (3)
C13—C14—C19—C18	−171.7 (3)	C11—N2—Zn1—N2^i^	−94.1 (3)
C18—C17—C20—O4	2.6 (6)		

Symmetry codes: (i) −*x*+1, *y*, −*z*+3/2.

### Hydrogen-bond geometry (Å, °)

**Table:**

*D*—H···*A*	*D*—H	H···*A*	*D*···*A*	*D*—H···*A*
O3—H3A···O2^ii^	0.82	1.74	2.538 (3)	162
O1W—H1B···O4^iii^	0.86 (3)	2.24 (2)	2.966 (4)	143 (3)
O1W—H1A···O2	0.85 (3)	2.00 (2)	2.808 (4)	159 (4)

Symmetry codes: (ii) *x*+1/2, *y*−1/2, *z*; (iii) −*x*+1, −*y*, −*z*+2.

## References

[bb1] Bruker (2001). *SADABS* Bruker AXS Inc., Madison, Wisconsin, USA.

[bb2] Bruker (2007). *SMART* and *SAINT* Bruker AXS Inc., Madison, Wisconsin, USA.

[bb3] Che, G.-B., Liu, H., Liu, C.-B. & Liu, B. (2006). *Acta Cryst.* E**62**, m286–m288.

[bb4] Chen, R., Guo, F. & Meng, F. (2008). *Acta Cryst.* E**64**, m761. 10.1107/S160053680801012X PMC2961536 21202454

[bb5] Lehn, J. M. (1990). *Angew. Chem. Int. Ed. Engl.* **29**, 1304–1305.

[bb6] Sheldrick, G. M. (2008). *Acta Cryst.* A**64**, 112–122. 10.1107/S0108767307043930 18156677

[bb7] Zang, S.-Q., Yang, S., Li, Y.-Z., Ni, Z.-P. & Meng, Q.-J. (2006). *Inorg. Chem.* **45**, 174–180. 10.1021/ic051502m 16390053

